# Detailed investigations of the influence of catalyst packing porosity on the performance of THAI-CAPRI process for in situ catalytic upgrading of heavy oil and bitumen

**DOI:** 10.1007/s13202-021-01327-7

**Published:** 2021-10-18

**Authors:** Muhammad Rabiu Ado

**Affiliations:** Department of Chemical Engineering, College of Engineering, King Faisal University, P.O. Box: 380, Al-Ahsa, 31982 Kingdom of Saudi Arabia

**Keywords:** In situ catalytic upgrading, Toe-to-heel air injection (THAI), In situ combustion (ISC), Enhanced oil recovery (EOR), Reservoir simulation, Heavy oil/bitumen/tar sand

## Abstract

Heavy oils and bitumen are indispensable resources for a turbulent-free transition to a decarbonized global energy and economic system. This is because according to the analysis of the International Energy Agency’s 2020 estimates, the world requires up to 770 billion barrels of oil from now to year 2040. However, BP’s 2020 statistical review of world energy has shown that the global total reserves of the cheap-to-produce conventional oil are roughly only 520.2 billion barrels. This implies that the huge reserves of the practically unexploited difficult-and-costly-to-upgrade-and-produce heavy oils and bitumen must be immediately developed using advanced upgrading and extraction technologies which have greener credentials. Furthermore, in accordance with climate change mitigation strategies and to efficiently develop the heavy oils and bitumen resources, producers would like to maximize their upgrading within the reservoirs by using energy-efficient and environmentally friendly technologies such as the yet-to-be-fully-understood THAI-CAPRI process. The THAI-CAPRI process uses in situ combustion and in situ catalytic reactions to produce high-quality oil from heavy oils and bitumen reservoirs. However, prolonging catalyst life and effectiveness and maximizing catalytic reactions are a major challenge in the THAI-CAPRI process. Therefore, in this work, the first ever-detailed investigations of the effects of alumina-supported cobalt oxide–molybdenum oxide (CoMo/γ-Al_2_O_3_) catalyst packing porosity on the performance of the THAI-CAPRI process are performed through numerical simulations using CMG STARS. The key findings in this study include: the larger the catalyst packing porosity, the higher the accessible surface area for the mobilized oil to reach the inner coke-uncoated catalysts and thus the higher the API gravity and quality of the produced oil, which clearly indicated that sulphur and nitrogen heteroatoms were catalytically removed and replaced with hydrogen. Over the 290 min of combustion period, slightly more oil (i.e. an additional 0.43% oil originally in place (OOIP)) is recovered in the model which has the higher catalyst packing porosity. In other words, there is a cumulative oil production of 2330 cm^3^ when the catalyst packing porosity is 56% versus a cumulative oil production of 2300 cm^3^ in the model whose catalyst packing porosity is 45%. The larger the catalyst packing porosity, the lower the mass and thus cost of the catalyst required per m^3^ of annular space around the horizontal producer well. The peak temperature and the very small amount of produced oxygen are only marginally affected by the catalyst packing porosity, thereby implying that the extents of the combustion and thermal cracking reactions are respectively the same in both models. Thus, the higher upgrading achieved in the model whose catalyst packing porosity is 56% is purely due to the fact that the extent of the catalytic reactions in the model is larger than those in the model whose catalyst packing porosity is 45%.

## Introduction

As the global attention is being heavily focused on the decarbonization of the world’s energy and economic systems, fossil fuels are still needed to provide an essentially turbulent-free transition. Still, as shown by the International Energy Agency (IEA) predictions, fossil fuels are expected to continue to meet a major share of the overall world’s energy demand up to the year 2040 (International Energy Agency, 2018). In addition to energy usage, this is also in order to continue to supply the surging demands in the petrochemicals and transportation (mainly planes, ships, and trucks) sectors. Analysing the latest projections by the IEA (2020) which factored in the highly destabilizing impact of COVID-19 pandemic to the energy, especially oil, industry, reveals based on estimation that by 2040, if the COVID-19 crisis is brought under control by 2021, and if governments around the world keep their current energy policies, the world would have consumed a total of 770 billion barrels of oil (International Energy Agency, 2020). However, according to the latest British Petroleum (2020) Statistical Review of World Energy 2020, the global total proved reserves of light (conventional) oils and heavy oils and bitumen/tar sand (unconventional oils) are 1734 billion barrels as at the end of 2019. The former accounts for only roughly 30%, whilst the latter accounts for the remaining 70% of the total (Elahi et al., 2019; Guo et al., 2016; Liu et al., 2019). Putting all the above values into perspectives shows that the current worldwide proved total reserves of the easy-and-cheap-to-develop-and-produce conventional oils is 520.2 billion barrels and if all of it is to be consumed, it will only provide around 67.6% of the total amount of oil needed from now up to the year 2040. Simply put, the remaining 32.4% or 249.8 billion barrels of oil needed must come from other oil sources, and this is where the unconventional oils (i.e. heavy oils and bitumen/tar sand) come in. Heavy oils and bitumen contain high fraction of asphaltic molecules which comprise preasphaltene, asphaltenes, and resins. As a result, under native reservoir conditions, heavy oils and bitumen are dense with American Petroleum Institute (API) gravity of lower than 20° and they are very sticky with very large viscosities ranging from hundreds to millions of centipoise (cP) (Hein, 2017; Li et al., 2020; Zhang et al., 2019). Whilst heavy oils have some partial mobility, bitumen is virtually immobile at the typical reservoirs temperatures and pressures. Hence, these resources must be stimulated by altering their flow and in some cases their chemical properties to either enhance or provide mobility. Different techniques, such as surface mining where the oil is located at shallow depth, cold production where the oil has some mobility, steam injection, in situ combustion, fluids (e.g. nitrogen, carbon dioxide, light hydrocarbons, polymers, surfactants, alkaline, etc.) injection, electrical resistive or inductive heating, in situ catalytic upgrading process, etc., are used to produce oil from the heavy oils, tar sand and bitumen reservoirs (Guo et al., 2016; Li et al., 2020; Mokrys and Butler, 1993; Shah et al., 2010; Yuan et al., 2019). Among the different techniques, thermal processes for heavy oils and bitumen upgrading and recovery have been shown to exhibit higher recovery factors both at laboratory and during field trials, and even at commercial scale (Guo et al., 2016; Shah et al., 2010). This is primarily because the viscosities of heavy oils and bitumen decrease exponentially with increase in temperature. However, among the thermal enhanced oil recovery (EOR) processes, the steam-injection-based processes (i.e. the steam-assisted gravity drainage (SAGD), the cyclic steam stimulation (CSS), and the steam flooding (SF)) have been shown to suffer from substantial wellbore heat losses, to result in the release of large amount of carbon dioxide (CO_2_) from the steam generation, to exhibit a lower recovery factor compared to other thermal EOR processes, to have limited applicability to reservoirs with large oil pay thickness, to handle large amount of wastewater, to be negatively affected by reservoir heterogeneities such as shale barriers, and to not achieve appreciable degree of heavy-to-light oil upgrading within the reservoir compared to their counterparts, namely the in-situ-combustion-type processes (Gates, 2010; Gates and Larter, 2014; Ma and Leung, 2020; Mokrys and Butler, 1993; Shah et al., 2010; Shi et al., 2017; Zhao et al., 2014, 2013).

The in situ combustion thermal EOR processes (i.e. the conventional in situ combustion (ISC) and the toe-to-heel air injection (THAI)) have been shown to overcome all the disadvantages of the steam-based processes (Greaves et al., 2008). Additionally, they achieve substantial underground upgrading of the heavy oils, tar sand and bitumen in such a way that chemically and physically altered pipeline-able light oils are produced. They also hold huge economic promise (Liu et al., 2021) and from the latest field operations information reported in this and last years, the ISC and the THAI process are very efficient (Sharma et al., 2021; Turta et al., 2020; Wei et al., 2020b). ISC, however, is shown to suffer from gravity segregation and thus excessive gas override, and the mobilized upgraded oil has to travel over hundreds of metres before it can reach the vertical production well. The THAI process on the other hand falls into the category of the so-called "short-distance displacement" processes (Turta and Singhal, 2004), and laboratory studies have shown it to exhibit very high recovery factors of up to 85% of oil originally in place (OOIP) and to provide upgrading by up to 8.2° API (Greaves et al., 2008, 1999; Xia and Greaves, 2006). The THAI process has been extensively studied at laboratory (Greaves et al., 2008; Turta and Singhal, 2004; Xia et al., 2002a, 2002b, 2002c; Xia and Greaves, 2006, 2002; Xia et al., 2002a; Zhao et al., 2021, 2018), via laboratory-scale and field-scale numerical simulations (Ado, 2021, 2020a, 2020b, 2020c; Ado et al., 2019; Greaves et al., 2012a, 2012b, 2012c; Rabiu Ado et al., 2018, 2017) and at pilot and semi-commercial scales (Petrobank, 2009, 2008; Touchstone, 2016, 2015; Turta et al., 2020; Wei et al., 2020a, 2020b). These studies have shown that when compared against other heavy oils recovery techniques, the THAI process has the following realizable potentials: (i) energy-self-sufficiency when waste heat is used to run utilities, (ii) having minimal surface-footprint as no surface upgrading facilities are needed, (iii) potential for being carbon-capture-ready so that the captured CO_2_ can be sequestered or be used for EOR, etc. Additionally, the other main advantage of the THAI process is the ease with which a bed of catalyst can be emplaced around the horizontal producer (HP) well to provide additional upgrading inside the reservoir. This, thus, results in essentially converting the reservoir into an underground catalytic reactor. The downhole catalytic upgrading process is referred to as Catalytic Add-on PRocess In situ (CAPRI™), and when combined with the THAI process, it is known as THAI-CAPRI. The THAI-CAPRI process involves packing an industrial hydro-processing catalysts, such as alumina-supported cobalt oxide–molybdenum oxide (CoMo/γ-Al_2_O_3_), alumina-supported nickel–molybdenum oxide (NiMo/γ-Al_2_O_3_,), and alumina-supported zinc oxide–copper oxide (ZnCu/γ-Al_2_O_3_), around a horizontal producer well to remove impurities, such as sulphur, nitrogen, heavy metals, and lock them permanently inside the reservoir. This, therefore, results in achieving further upgrading of the otherwise difficult-to-produce unconventional oils. In the THAI-CAPRI process, the partially upgraded THAI oil which is formed due to heat from combustion (i.e. due to thermal cracking) serves as the reactant that must contact the catalyst bed for catalytic upgrading reactions to take place. The THAI-CAPRI process has the advantages, among others, of providing easily transportable oil, lowering surface upgrading requirements, providing environmentally friendly operations, etc.

Many laboratory and a simulation studies were and are being performed towards understanding the downhole or in situ catalytic upgrading of heavy oils, bitumen and tar sands (Abu et al., 2015; Abuhesa and Hughes, 2009; Ado et al., 2021; Cavallaro et al., 2008; Galukhin et al., 2017; Greaves et al., 2004; Hasan and Rigby, 2019; Karimian et al., 2018; Mehrabi-Kalajahi et al., 2021; Moore et al., 1999; Rabiu Ado, 2017; Shah et al., 2011; Weissman, 1997; Weissman et al., 1996; Xia and Greaves, 2001; Xia et al., 2002b; Yuan et al., 2021). Xia and Greaves (2001) studied downhole catalytic upgrading of Wolf Lake heavy oil. Apart from 10° API increase due to thermal upgrading, they achieved a further upgrading by 4 to 7° API using nickel–molybdenum (NiMo) or cobalt–molybdenum (CoMo) HDS catalysts. Substantial viscosity reduction from the partially upgraded THAI oil was observed. Application of the THAI-CAPRI process to Lloydminster heavy oil resulted in an overall upgrading by 11.6° API (Xia et al., 2002b). The highest upgrading was obtained with regenerated CoMo HDS catalyst. Similarly, the reduction in viscosity was also very substantial. An analysis of the extent of upgrading and investigation of the properties of the upgraded oil from three-dimensional (3D) combustion cell THAI-CAPRI experiment was carried out by Greaves et al. (2004). Quantitatively, they showed that the sulphur, nitrogen, and metals content of the produced oil were significantly reduced compared to their concentrations in the original oil prior to being upgraded. The overall oil quality in terms of API gravity and viscosity was substantially improved. However, the fate of the removed sulphur, nitrogen, and the heavy metals has not been determined. Moreover, the effect of fluid flow and coke deposition on catalyst was not studied. However, it is noted that the amount of residual solid carbon deposited was increased by 3 to 6 times during the THAI-CAPRI process (Greaves et al., 2004) compared to that obtainable in the THAI process alone. Another experiment conducted by Cavallaro et al. (2008) installed a heated bed of catalyst upstream of a producer well in a combustion tube. The presence of the catalyst bed resulted in considerable delay in the oil production. Once oil production was started and the combustion front approached the catalyst bed, external heaters were used to heat the bed to a temperature of 330 °C over a specific period of time. An increased oil production rate was observed which was thought to be the result of the external heating. After the bed became effective, a reduction in heteroatoms in the produced oil was observed. As in the case of the 3D combustion cell experiments reviewed earlier, substantial viscosity reduction and increase in API gravity were achieved with the NiMo catalyst. One major source of concern during that experiment was the observance of excessive pressure drop which could be due to catalyst packing porosity blockage as a result of increased coke deposition. This explanation is supported by the fact that the reported fuel concentrations for combustion tube experiments with and without catalyst bed are considerably different with that of the former far higher than that in the latter. It, therefore, became difficult to conclude whether the achieved substantial upgrading is solely due to catalytic action or that the low oil mobility and high-temperature combustion resulted in significant thermal cracking. In further experimental studies, carried out by Shah et al. (2011), on the optimization of the THAI-CAPRI process, severe limitations to the life of the catalyst were observed as a result of coke deposition on the catalyst surface. The experiment was run in a catalytic reactor operated at pressure of 20 to 60 bar and temperature of 380 to 500 ^o^C in the presence of hydrogen gas. Partially upgraded heavy oil from THAI pilot testing with API gravity of 13° was used. At optimum temperature of 420 °C, only a maximum of 3°API upgrading was obtained with catalyst life of roughly 3.25 days. They concluded that coking, which leads to catalyst pore blockage, occurred at very early stage of the process while the upgrading was taking place. Also, it was observed during the experiment that substantial pressure drop, which was attributed to blockage due to severe catalyst coking, occurred. Apart from the laboratory studies of the in situ catalytic upgrading, a single pilot test of the THAI-CAPRI process was undertaken by Petrobank Energy Resources LTD. as part of their Whitesands project, in Alberta, Canada, in 2008 (Petrobank, 2008). Although the project achieved some additional upgrading compared to the purely thermal effect in the THAI process alone, it was deemed insufficient to continue with it.

In all the in situ catalytic processes studies outlined earlier, severe catalyst pore blockage due to coking has been identified as one of the major problems affecting the process’s performance (Cavallaro et al., 2008; Greaves et al., 2004; Shah et al., 2011). Furthermore, it is worth pointing out that these studies are not the first to show that coking deactivates catalyst during hydro-treating. An extensive review by Bartholomew (2001) shows that coking during hydro-treating results in very severe catalyst deactivation to the extent that catalyst regeneration to regain activity might not be a viable option and costly replacement might have be made. Given the challenging practical difficulties and costly nature of maintaining horizontal producer (HP) well, ways of maximizing catalyst life and thus effectiveness during the operation of the in-situ-catalytic-upgrading-type processes must be fully delineated prior to any field deployment. This is where this first-of-a-kind study comes in. The cheapest and most straight forward way to discern the physicochemical processes on the catalyst coking in the THAI-CAPRI process is through the use of reservoir numerical simulations. The first simulation study of the THAI-CAPRI process was conducted by Rabiu Ado (2017) where a catalyst packing porosity of 45%, which lies in between the 44% and 45.1% reported from experimental studies performed by Abu et al. (2015), was used. Then, using the Rabiu Ado’s (2017) THAI-CAPRI numerical model, Hasan and Rigby (2019) conducted a numerical simulation studies where they reported the effect of the catalyst packing porosity on the cumulative oil production and the peak temperature. However, their study has three limitations: (1) They only investigated packing porosities of 44%, 44.55%, and 45.1%, and that means the differences in the packing porosities are so small that no pronounced effect was observed both with the cumulative oil production and the peak temperature, (2) they only considered two parameters (i.e. cumulative oil production and peak temperature) when investigating the effects that these limited ranges of packing porosities have on the two selected parameters, and (3) they did not provide justifications as to why the critical parameters in studying the effect of catalyst packing porosity, namely degree of upgrading (API gravity), oxygen production, and hydrogen sulphide production, are not considered.

Given the above findings from the literature and the critical importance of preserving and prolonging catalyst life in situ the reservoir, the aim of this work is to present and analyse how the performance of the THAI-CAPRI process is affected by the catalyst packing porosity. For that, a thoroughly well-tested THAI-CAPRI numerical model developed using the computer modelling group (CMG) thermal reservoir simulator, STARS, is used. Two numerical models which have identical input parameters but differ in terms of catalyst packing porosity were developed. The first model, namely P45, has a catalyst packing porosity of 45%, whilst model P56 has a catalyst packing voidage of 56%. As during surface catalytic cracking, the presence of hydrogen is a necessary requirement for in situ catalytic upgrading of heavy oils and bitumen. However, previous findings from multiple studies and from field data (Abu et al., 2015; Hajdo et al., 1985; Kapadia et al., 2015, 2013, 2011; Petrobank, 2008; Shah et al., 2010; Weissman, 1997) have shown that in situ combustion results in hydrogen formation, which is thought to be generated as a result of thermal cracking, aquathermolysis, water–gas shift, and coke gasification reactions. For example, BP Resources Canada LTD. reported from their in situ combustion pilot at Marguerite Lake, Alberta, Canada, that as high as 20 mol% hydrogen was consistently produced (Hajdo et al., 1985). Similarly, from the recent THAI process pilot in Whitesands, Canada, Petrobank Energy Resources LTD. reported that up to 8 mol% hydrogen was produced (Petrobank, 2008). However, in this study, the hydrogen was injected together with air which is in accordance with study conducted by Shah et al. (2011) in which they externally supplied the required hydro-treating hydrogen. The results of the investigations considered all the critical parameters required to assess the applicability and performance of the THAI-CAPRI process.

## Methodology of the numerical models construction

### Control volume dimensions and wells configuration

**Fig. 1:**
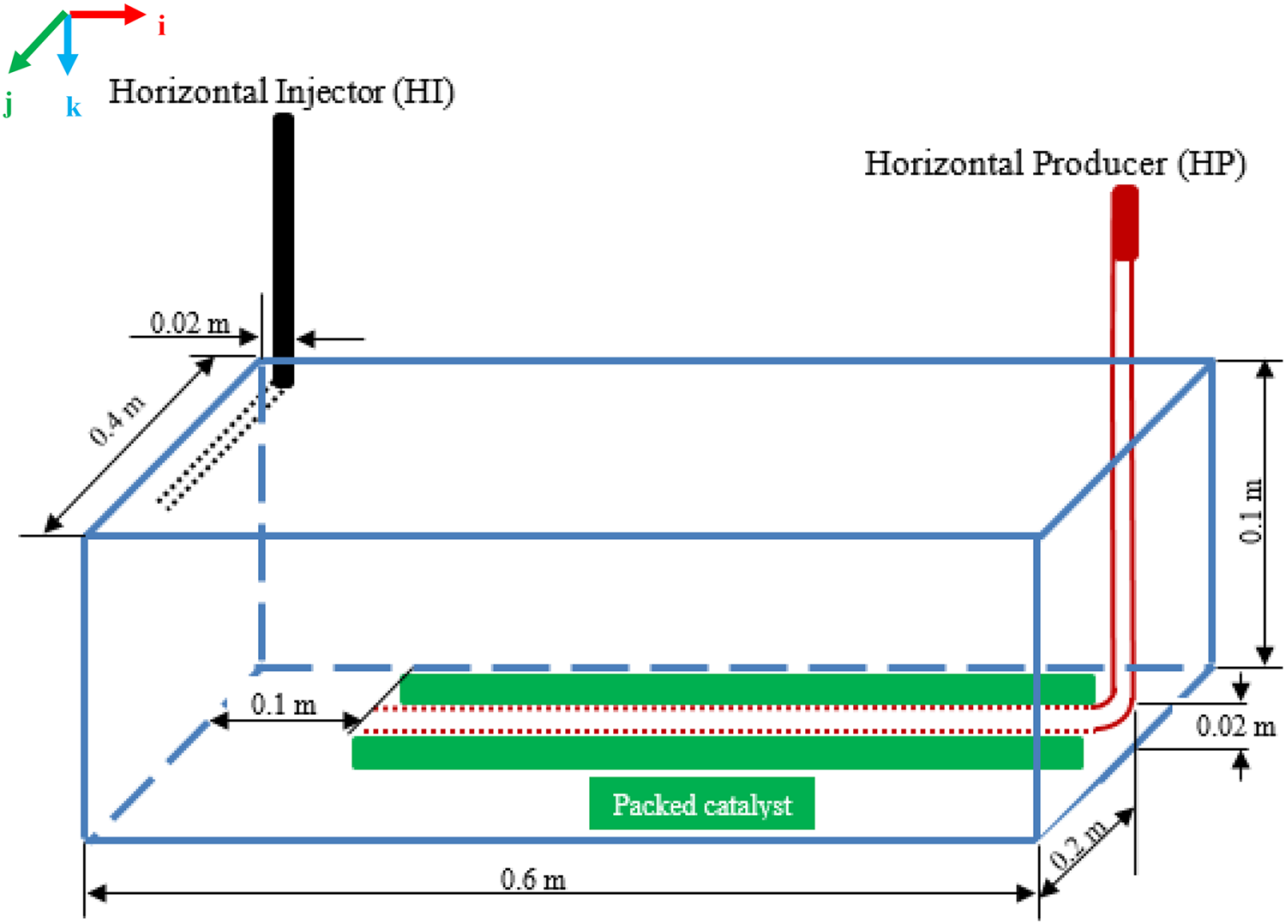
3D experimental-scale control volume of the THAI-CAPRI process with dimensions and wells configuration as used in both models P45 and P56 which have catalyst packing porosities of 45% and 56% respectively

In this work, experimental-scale dimensions, which are similar to those reported in previous works and upon which the THAI and THAI-CAPRI processes numerical models were validated (Ado, 2020d; Rabiu Ado, 2017; Rabiu Ado et al., 2017), are used to represent the 3-dimensional (3D) control volume of the THAI-CAPRI process (Fig. 1). The control volume has both a horizontal producer (HP) well, which is surrounded by a thick green layer as the annular catalyst layer, and a horizontal injector (HI) well which are arranged in a staggered line drive (SLD) configuration.

### Properties of reservoir rock and those of its fluids

**Fig. 2:**
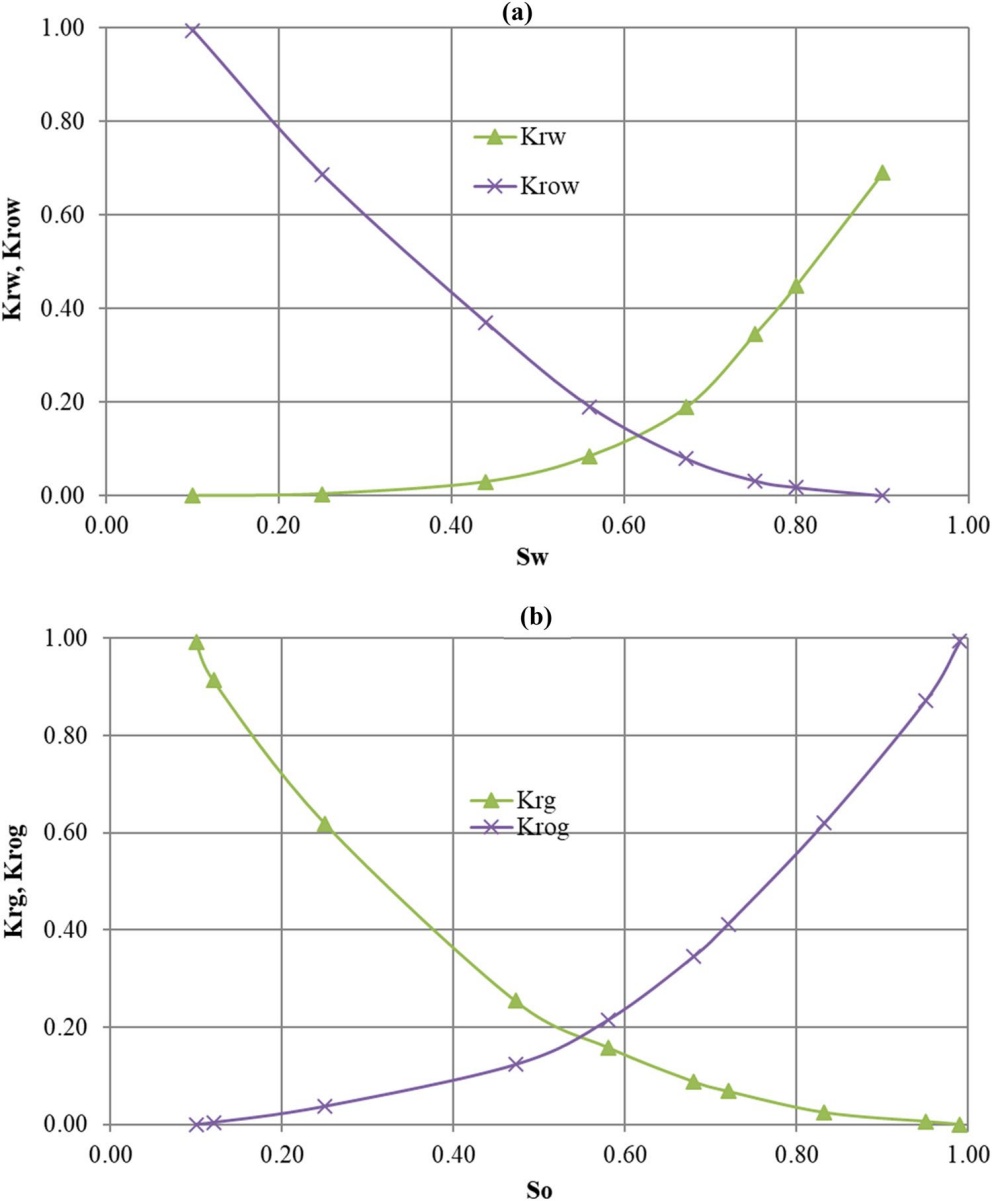
**a** Oil–water and **b** gas–oil relative permeability curves for the Athabasca bitumen reservoir rock respectively

The effective formation compressibility of the reservoir is 1.40 × 10^–5^ kPa^−1^. The horizontal and vertical absolute permeabilities are 11,500 mD and 3450 mD respectively. The thermal properties of the reservoir rock and those of its fluids are given in Table 1. Figure 2 shows the oil/water and gas/oil relative permeability curves for the Athabasca bitumen reservoir rock. All these input parameters are used in both models.

**Table 1:** Thermal properties of the reservoir rock and those of its fluids

Thermal properties of the reservoir rock and its fluids	Values
Heat capacity of rock	2600 kJ m^−3^ °C^−1^
Thermal conductivity of rock	458.3333 J m^−1^ min^−1^ °C^−1^
Oil thermal conductivity	7.9861 J m^−1^ min^−1^ °C^−1^
Gas thermal conductivity	3.4722 J m^−1^ min^−1^ °C^−1^
Water thermal conductivity	37.1528 J m^−1^ min^−1^ °C^−1^

### Reservoir initial and boundary conditions

The initial oil, water, and gas saturations are 0.85, 0.15, and 0.0 respectively, and the porosity and initial reservoir temperature are 0.34 and 20 °C respectively (Rabiu Ado, 2017). A no-flow boundary condition was assigned to the whole control volume except via the HI and HP wells. Overburden and underburden conductive heat loss parameters were also specified for the models in accordance with previously validated models (Ado, 2020d; Rabiu Ado, 2017). The producer back pressure was set at absolute pressure of 80 bar, and thus, the whole control volume was maintained at that pressure. The inlet zone of the HI well was electrically pre-heated for 30 min prior to gas injection. The injected gas is made up of hydrogen-to-air ratio of 1:4. Immediately after the pre-ignition heating cycle (PIHC), the gas was first injected at a flux of 15 m^3^ m^−2^ h^−1^ (i.e. at injection rate of 10,000 cm^3^ min^−1^) for a duration of 160 min (i.e. up to 190 min from the start of the process). Thereafter, the injection flux was increased by a factor of 4/3 (i.e. to 20 m^3^ m^−2^ h^−1^ (i.e. at injection rate of 13,333 cm^3^ min^−1^)) for the rest of the process time (i.e. up to 320 min).

### Pressure, density, temperature, and phase properties

**Fig. 3:**
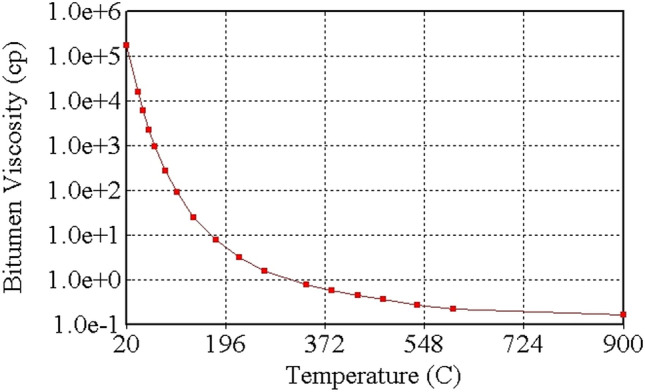
Athabasca bitumen viscosity as function of temperature

To simulate the THAI-CAPRI process, the pressure, density, and temperature (PρT) parameters used, which are for the Canadian Athabasca tar sand/bitumen and for the CAPRI-produced oil from the Athabasca tar sand/bitumen, are similar to those reported in previous works in Ado (2020d) and Rabiu Ado (2017) (pp. 297–299) respectively as can be seen in Table 2 and Table 3 respectively. The PρT properties in Table 3 were obtained based on validating experimental results using Peng–Robinson equation of state in Aspen Hysys. To account for phase changes, Wilson equation was used to estimate the phase equilibrium K-values of the THAI-CAPRI oil pseudo-components and hence are reported as functions of both temperature and pressure in the previous work by Rabiu Ado (2017) (pp. 300–301). The viscosity of each oil pseudo-component as a function of temperature is also given in previous work by Rabiu Ado (2017) (pp. 299–300). Therefore, these are not repeated here to avoid duplications and consequently, the reader is referred to the provided reference. However, the viscosity of the Athabasca bitumen as a function of temperature is given in Fig. 3.

**Table 2:** Pressure, density, temperature, acentric factor, molecular mass, and composition of the oil pseudo-components making up the Canadian Athabasca bitumen

Athabasca bitumen pseudo-components	Split (mol%)	RMM (g/mol)	P_c_ (kPa)	T_c_ (°C)	ρ (kg/m^3^)	Acentric factor ω
LITE oil	36.47	170.00	2305.95	425.16	903.80	0.48
HEAV oil	63.53	878.00	1031.29	780.00	1012.07	1.45

**Table 3:** Pressure, density, temperature, acentric factor, molecular mass, and composition of the CAPRI-produced oil pseudo-components from the Canadian Athabasca bitumen

CAPRI-produced oil pseudo-components	Split (mol%)	RMM (g/mol)	P_c_ (kPa)	T_c_ (°C)	ρ (kgm^−3^)	Acentric factor ω
LUO	20.31	128.01	2448.61	353.12	776.53	0.39
HUO	79.69	252.50	1523.46	502.19	850.48	0.70

### Kinetics scheme and parameters

Apart from the thermal cracking and combustion reactions typically required to simulate in-situ-combustion-type processes, catalytic reactions are also required when simulating the THAI-CAPRI process. The thermal cracking and combustion reactions and their kinetics parameters used in this work are the same as those in the validated ‘‘model G’’ in the previous work by Ado (2020d). The API gravity-based validated catalytic upgrading reactions kinetics scheme and parameters which are reported in the previous work (Ado et al., 2021) are used in this work. Thus, the coupled reactions that take place in the THAI-CAPRI process are shown below.

Thermal cracking reaction:

$${\text{Heavy oil}} \to {1}{\text{.6 Light oil + 46}}{\text{.6 Coke}}$$

Combustion reactions:

$$\begin{array}{*{20}c} {{\text{Heavy oil + 80 O}}_{{2}} \to {26}{\text{.7 H}}_{{2}} {\text{O + 68}}{\text{.7 CO}}_{{\text{X}}} } \\ {{\text{Light oil + 19 O}}_{{2}} \to {14}{\text{.5 H}}_{{2}} {\text{O + 11}}{\text{.8 CO}}_{{\text{X}}} } \\ {{\text{CH + 1}}{\text{.2 O}}_{{2}} \to {0}{\text{.5 H}}_{{2}} {\text{O + CO}}_{{\text{X}}} } \\ \end{array}$$

Catalytic hydrodesulphurization and hydrodenitrogenation reactions:

$$\begin{array}{*{20}c} {{\text{Heavy oil + 5}}{\text{.7470 H}}_{{2}} \to {3}{\text{.4770 UHO + 0}}{\text{.2925 H}}_{{2}} {\text{S + 0}}{\text{.0945 NH}}_{{3}} } \\ {{\text{Light oil + 1}}{\text{.6905 H}}_{{2}} \to {1}{\text{.3280 ULO + 0}}{\text{.0968 H}}_{{2}} {\text{S + 0}}{\text{.0055 NH}}_{{3}} } \\ \end{array}$$

### Catalyst properties and activation

In this work, alumina-supported cobalt oxide–molybdenum oxide (CoMo/γ-Al_2_O_3_) catalyst is used which is in parallel with studies by Shah et al. (2011) and Ado (2021). The properties of the catalyst, namely molecular mass, bulk density, and specific heat capacity, used in both models in this work are similar to those reported in previous work by Rabiu Ado (2017). What is varied, however, is the catalyst packing porosity and hence the catalyst loading. For model P45, the catalyst packing porosity was 45% which corresponds to a catalyst loading of 574.42 kg m^−3^ and for model P56, the packing porosity was 56% which corresponds to a catalyst loading of 457.41 kg m^−3^. These large ranges of porosities of 11% and of catalyst loading of 117.01 kg m^−3^ are big enough to observe the potential effects of the catalyst packing porosity on the performance of the THAI-CAPRI process. Therefore, this work is not about determining the optimum catalyst packing porosity. Instead, it is conducted to see if the catalyst packing porosity has any effect on the performance of the THAI-CAPRI process or not.

To incorporate the catalytic reactions in the CMG STARS, the molecular weight of the catalyst must be part of the input parameters. For the catalyst to catalyse the reactions, and for the reactions to take place in the catalyst annular layer, the CoMo/γ-Al_2_O_3_ catalyst must be added as both part of the reactants and products simultaneously, as exemplified by the mass-and-atom-balanced chemical equation below:

$$\begin{array}{*{20}c} {{\text{Heavy oil}}\;{ + }\;{5}{\text{.7470 H}}_{{2}} \;{ + }\;{\text{Catalyst}} \to {3}{\text{.4770 UHO}}\;{ + }\;{0}{\text{.2925 }}} \\ {{\text{H}}_{{2}} {\text{S}}\;{ + }\;{0}{\text{.0945 NH}}_{{3}} \;{ + }\;{\text{Catalyst}}{.}} \\ \end{array}$$

This way, it is ensured that no catalyst is consumed in the reactions and that the catalytic reactions only take place when the mobilized partially upgraded oil and hydrogen contact the catalyst. However, previous studies (Greaves et al., 2012c; Rabiu Ado et al., 2017) have shown that the temperature of the mobile oil zone (MOZ) where the catalytic reactions are envisaged to happen, and let alone that of the HP well location below the cold oil zone (COZ), is lower than the greater than 330 °C temperature required for the hydro-treating catalysts to be activated and thus for the catalytic reactions to take place. As a result, the concept of activation temperature, first introduced by Coats (1983), is used to account for the required temperature for the catalytic reactions to occur. A minimum temperature of 400 °C is assigned to be used to calculate the Arrhenius reaction rates constants at every location in the annular catalyst layer that has temperature of lower than 400 °C. This implies that any location along the annular catalyst layer that has temperature of greater than 400 °C will use its temperature to calculate the Arrhenius reaction parameter, k. This, overall, implies that the catalyst layer is simulated as if it is being externally heated to a temperature of at least 400 °C whilst catalytic reactions take place. This allows, for the first time ever, the overcoming of the limitations of the current commercial reservoir simulators, such as the CMG STARS. As future suggestions, the incorporation of annular catalyst layer and electrical inductive heating coil to surround the HP well must be carried out prior to any field deployment of the THAI-CAPRI process. To partly help with these investigations, a new unpublished study by Ado has studied the feasibility of electrically heating the HP well by surrounding it with an electrical inductive heating coil in the THAI process alone. This full field-scale study has clearly revealed that it is indeed possible to electrically heat the HP well but in a selectively sectional way such that the heating takes place in a toe-to-heel manner. It has also shown that there is added advantage to electrically heating the HP well because the cumulative oil recovery was increased by 28% compared to in the base case model. It has also shown that larger fraction of the length of the HP well is used for mobilized oil production compared to in the base case model, thereby implying that greater catalytic effects will certainly be achieved with this setting. Furthermore, the study has shown that the flow of mobilized oil is not impeded by the addition of electrical heating coils.

### Grid blocks sensitivity analysis and reservoir discretization

**Fig. 4:**
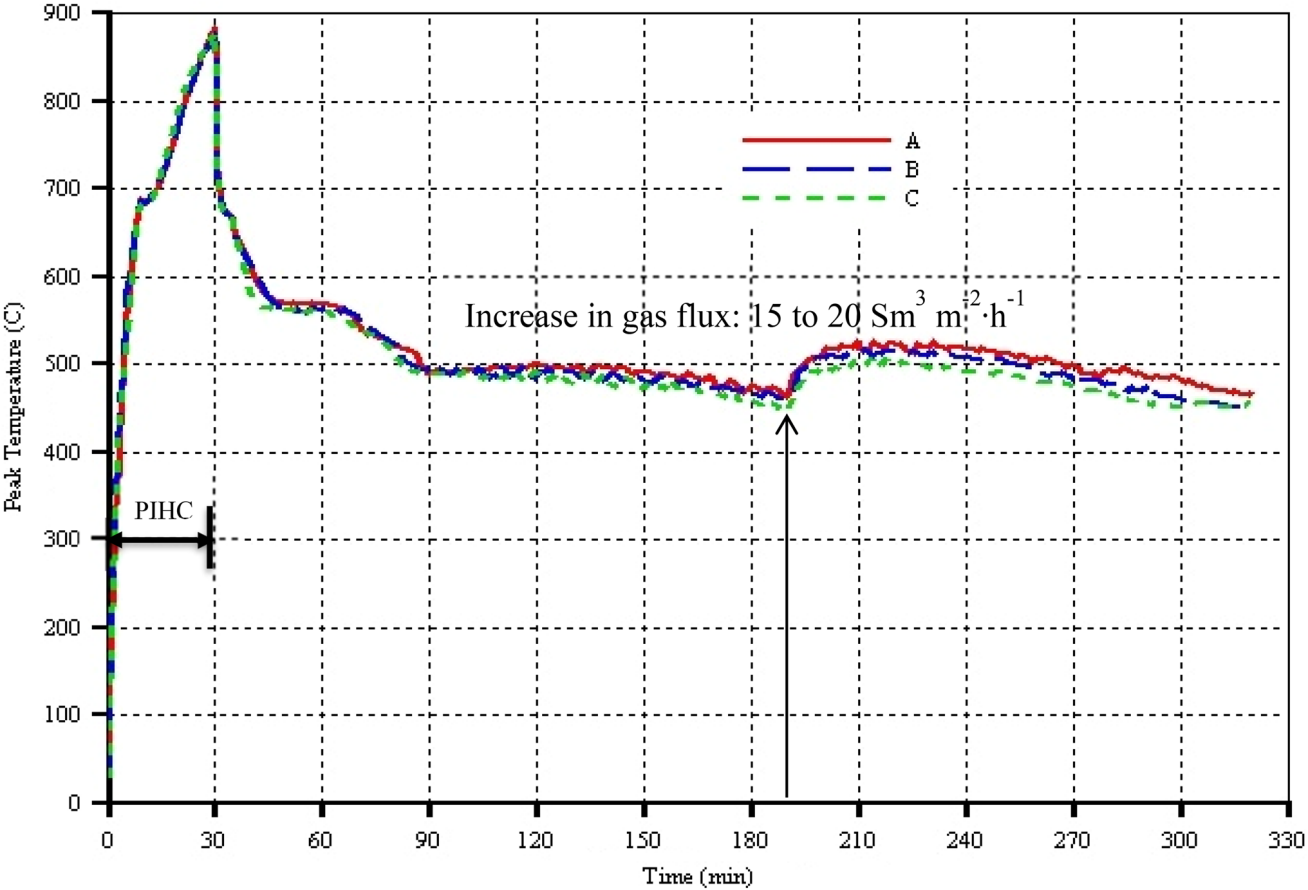
Peak temperature as function of time for the three different grid blocks sizes

**Fig. 5:**
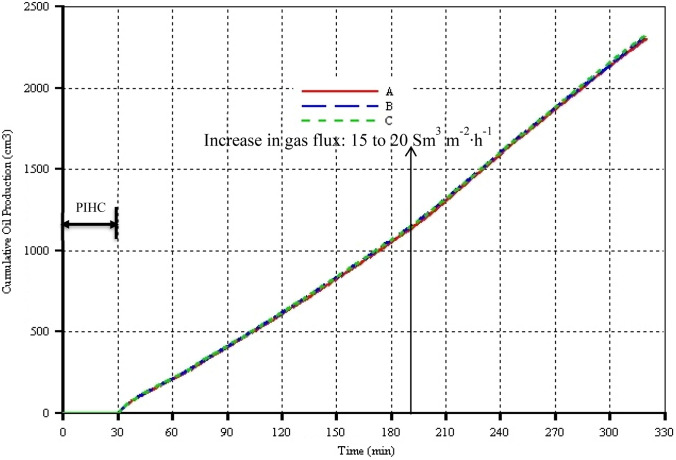
Cumulative oil production as function of time for the three different grid blocks sizes

In order to investigate the sensitivity of the simulation results to the grid blocks sizes, three numerical models of the THAI-CAPRI process with different grid blocks dimensions, as can be seen in Table 4, were run. Figure 4 shows the peak temperature as a function of time for the different models. It can be seen that regardless of the grid blocks sizes, the predicted peak temperatures are more or less the same. In Fig. 5, the cumulative oil production curves and thus the oil production rates are barely sensitive to the grid blocks sizes. Therefore, the control volume in this study was discretized into 30 in direction ***i*** by 19 in direction ***j*** by 9 in direction ***k***. For the latter two directions, variable thicknesses are used. As the combustion front thickness is around 2.5 cm, to capture it is full Physics, each grid block was further divided into 3 sub-grids in direction ***i***. Hence, the overall number of grid blocks + discretized wellbore is 19,900. The thickness of the annular catalyst bed is 0.5 cm. The numerical models studies are based on the Canadian Athabasca bitumen or tar sand properties. The numerical simulations were performed using computer modelling group (CMG) commercial thermal reservoir simulator, STARS.

**Table 4:** THAI-CAPRI models, grid blocks dimensions, and approximate total number of grid blocks of the reservoir plus that of the discretized wellbore model

THAI-CAPRI model	Grid block dimensions (**i** × **j** × **k**) (mm)	Number of grid blocks including those of the discretized wellbore model
A	6.67 × 23.13 × 14.00	19,900
B	6.67 × 11.56 × 14.00	32,860
C	6.67 × 7.71 × 14.00	45,820

## Results and discussion

Prior to analysing and discussing the most critical parameters determining the success or otherwise of the THAI-CAPRI process, it is worth pointing out at this stage that the two models were respectively run for a period of 320 min. This is because the 320 min are enough to observe any influence of the catalyst packing porosity on the performance of the THAI-CAPRI process. More so, it is also due to the fact that steady state condition is achieved within 50 min after the start of gas injection and initiation of the combustion.

### Oil production rate

**Fig. 6:**
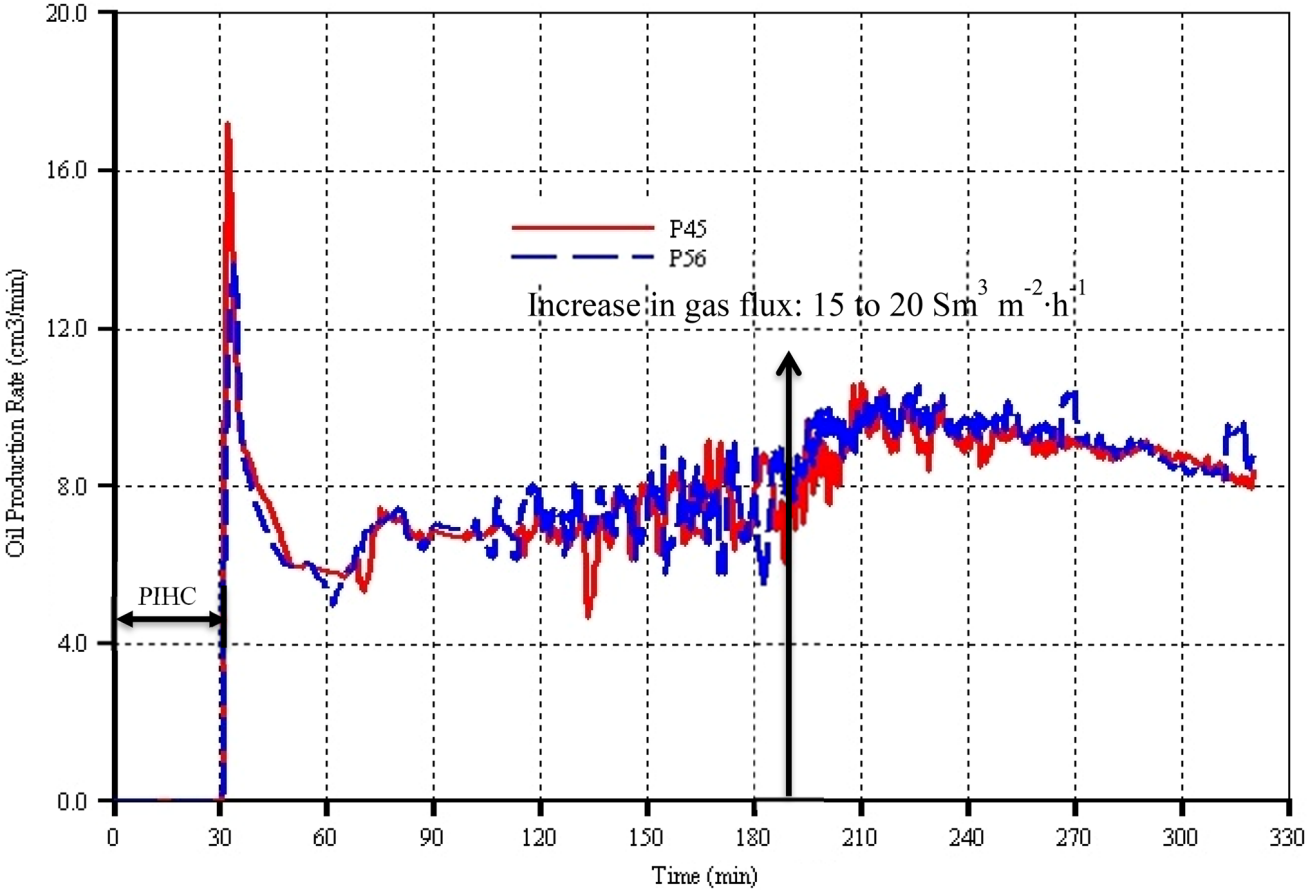
Oil production rate as function of process time for models P45 and P56 which have catalyst packing porosities of 45% and 56% respectively

**Fig. 7:**
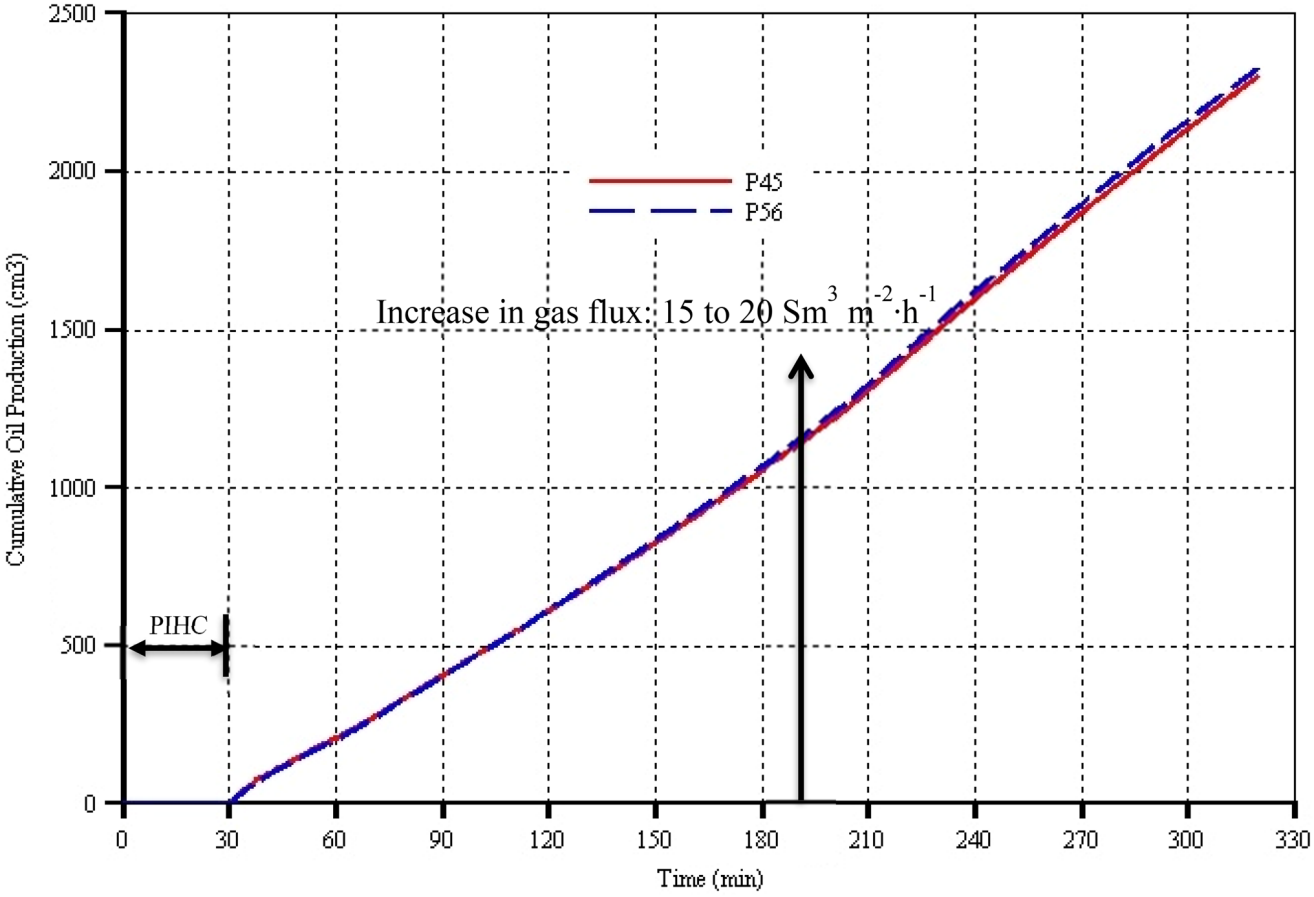
Cumulative oil production as function of process time for models P45 and P56 which have catalyst packing porosities of 45% and 56% respectively

Figure 6 shows that in both models, there is no oil production during the 30 min of the pre-ignition heating cycle (PIHC) period. The delay in oil production can be attributed to the very high pressure (i.e. 80 bar) at which the process is operated. Comparison with the validated THAI-only model G, which is reported in previous work by Ado (2020d), notwithstanding that it did not contain the catalytic reactions and the catalyst bed, revealed that when the process was operated at an absolute pressure of 2 bar, oil production began 15 min after the start of the electrical pre-heating. This 15-min delay, therefore, is not due to the presence of catalyst bed because the catalyst packing porosity of either of the two models (i.e. either of P45 or P56) is far higher than the reservoir 34% porosity and previous results show that the electrical pre-heating does not result in coke formation near the horizontal producer (HP) well. Coke is only formed at the top part of the reservoir in the vicinity of the horizontal injector (HI) well, which is one of the goals of the pre-heating. After about 4 min from the commencement of gas injection and initiation of combustion, oil production began in each model, peaking almost instantaneously to 17.5 cm^3^ min^−1^ and 14 cm^3^ min^−1^ in models P45 and P56 respectively (Fig. 6). Around 3 to 4 min afterwards, there is a very sharp decrease in the oil production rates in all the two models, reaching a value of roughly 10 cm^3^ min^−1^ (Fig. 6). These sudden peaking trends in the oil production rates are caused by releasing the pressure that was built-up by the light oil components from thermal cracking around the HI well and by gas pressure sweeping and forcing the already mobilized oil into the HP well and thus establishing fluid communication between the HI and the HP wells. And the subsequent rapid declining trends are due to the fact that the pressure was already released and that the injected gas has absorbed heat and cooled down the HI region of the reservoir, reducing the peak temperature as can be seen in Fig. 10. To minimise the effect of heat removal by injected and flue gases, a newly published field-scale THAI process study by Ado (2021) has shown that injecting pure oxygen in place of air resulted in increase in oil recovery by up to 3.85% OOIP (per cent oil originally in place). Furthermore, the study has shown that presence of nitrogen does not only impede the flow of mobilized oil into the HP well but also does consume otherwise useful heat. Therefore, these findings will be incorporated in future designs of the THAI-CAPRI process. From the neighbourhood of 38 min onwards, the oil production rates continue to decline but at slower rates, reaching minimums of 6 cm^3^ min^−1^ and 5.5 cm^3^ min^−1^ in models P45 and P56 respectively at 60 min (Fig. 6). This period in either model corresponds to that when the heat from combustion is continued to be used to heat up the incoming gas and that the heat has not been transferred far enough into the downstream mobile oil zone (MOZ) to effect further oil mobilization. That can be seen in Fig. 10 in which the peak temperatures continued to slowly decline until they reached 580 °C at 45 min and thereon remaining constant up to 68 min. Understanding this is very important, especially with regard to the control and stability of combustion front and maintenance of steady oil production. Therefore, this can be referred to as transient period and gas injection must not be ramped up since the combustion front is not yet stable and has not yet fully developed. From 60 to 80 min (Fig. 6), the oil rates in all the two models increase reaching a value of 7.5 cm^3^ min^−1^ before slightly declining and remaining at average constant overlapping rates. The 80–190-min period in either models indicates steady-state operation and that the combustion front is fully stable which are manifested in the cumulative oil production curves having constant overlapping slopes (see Fig. 7) and also in the coinciding peak temperatures (see Fig. 10) remaining constant during most of the period. The increase in gas injection flux from 15 to 20 Sm^3^ m^−2^ h^−1^ at 190 min resulted in steady increase in oil production rates in both models, reaching maximums at 220 min before decreasing steadily thereafter. These declining trends are due to the decrease in the quantity of the oil that can be mobilized and produced. As a result, the oil production has entered what Greaves et al. (2012b) referred to as ‘declining phase’, a time beyond which even increase in air injection rate will not result in increase in oil production rate. Correspondingly, over similar period, the overlapping peak temperatures (Fig. 10) in both models also increased steadily before later turning into declining trends. The manifestation of these increases can be seen in the change in the slopes of the cumulative oil production curves. Generally, throughout the combustion time, there is mostly an overlap between the predictions by model P45 and model P56, with the latter slightly high than the former in some other times. On the overall however, it can be argued that the oil production rate is only marginally affected by the catalyst packing porosity. However, with respect to value addition, more performance parameters must be accounted for and accordingly understood.

### Cumulative oil production

**Fig. 8:**
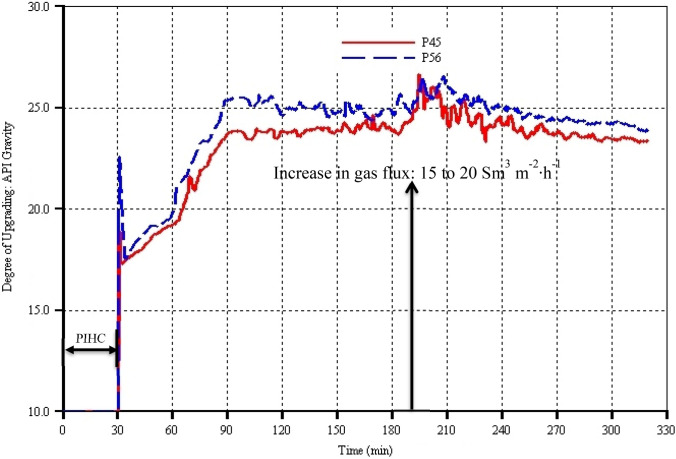
Degree of upgrading in terms of American Petroleum Institute (API) gravity as function of process time for models P45 and P56 which have catalyst packing porosities of 45% and 56% respectively

In accordance with the observations made with the regard to the oil production rates (Fig. 6), Fig. 7 shows that from 30 to 60 min there are slight variations in the slopes of the coinciding cumulative oil production curves of the two models. Afterwards and just prior to the increase in the gas injection flux, it can be seen that the nearly overlapping cumulative oil production curves have approximately constant slopes, indicating that the average oil production rates are the same and constant. However, there is a slight divergence in the two curves as 190 min is approached, with model P56 slightly lying above P45 (Fig. 7). On increasing the gas injection rate, the two models dynamically responded showing, though marginally, increase in oil production rates and thus the slopes of the cumulative oil production curves. However, the divergence between the two models increases revealing that the lying of model P56 curve slightly above the model P45 curve is more visible after the increase in the gas injection flux from 15 to 20 Sm^3^ m^−2^ h^−1^ (Fig. 7). Consequently, cumulatively, slightly more oil is produced, and thence recovered, in model P56 by around 30 cm^3^ compared to that in model P45 (Fig. 7). This is justified given that model P56 has generally higher API points (as can be seen in Fig. 8), and hence, higher amount of light components, compared to in model P45. On the overall, however, it is concluded that the cumulative oil production is only marginally affected by the catalyst packing porosity. Moreover, these conclusions, which undoubtedly come out from this study, based on the oil production rates and cumulative oil production can be fully supported and validated by findings in previous studies (Ado et al., 2021). Observing the actual location of the mobilized oil flux vectors, which can be seen to overlap with thermal cracking and the coke-deposited zones, reveals that mobilized oil flow onto the surface of the catalyst can indeed be impeded by deposited coke. However, this finding is only valid at experimental scale (i.e. in both physical laboratory experiments and experimental-scale simulations). Findings from field-scale simulations indicated that significant amount of mobilized oil is actually produced, for most of the time, via the toe of the HP well which is, also for most time, behind the coke-deposited zones. Therefore, it is highly likely that the oil production rates will be highly rather than slightly impeded by coke. Therefore, further investigations at field scale are warranted.

### Degree of upgrading (API gravity)

**Fig. 9:**
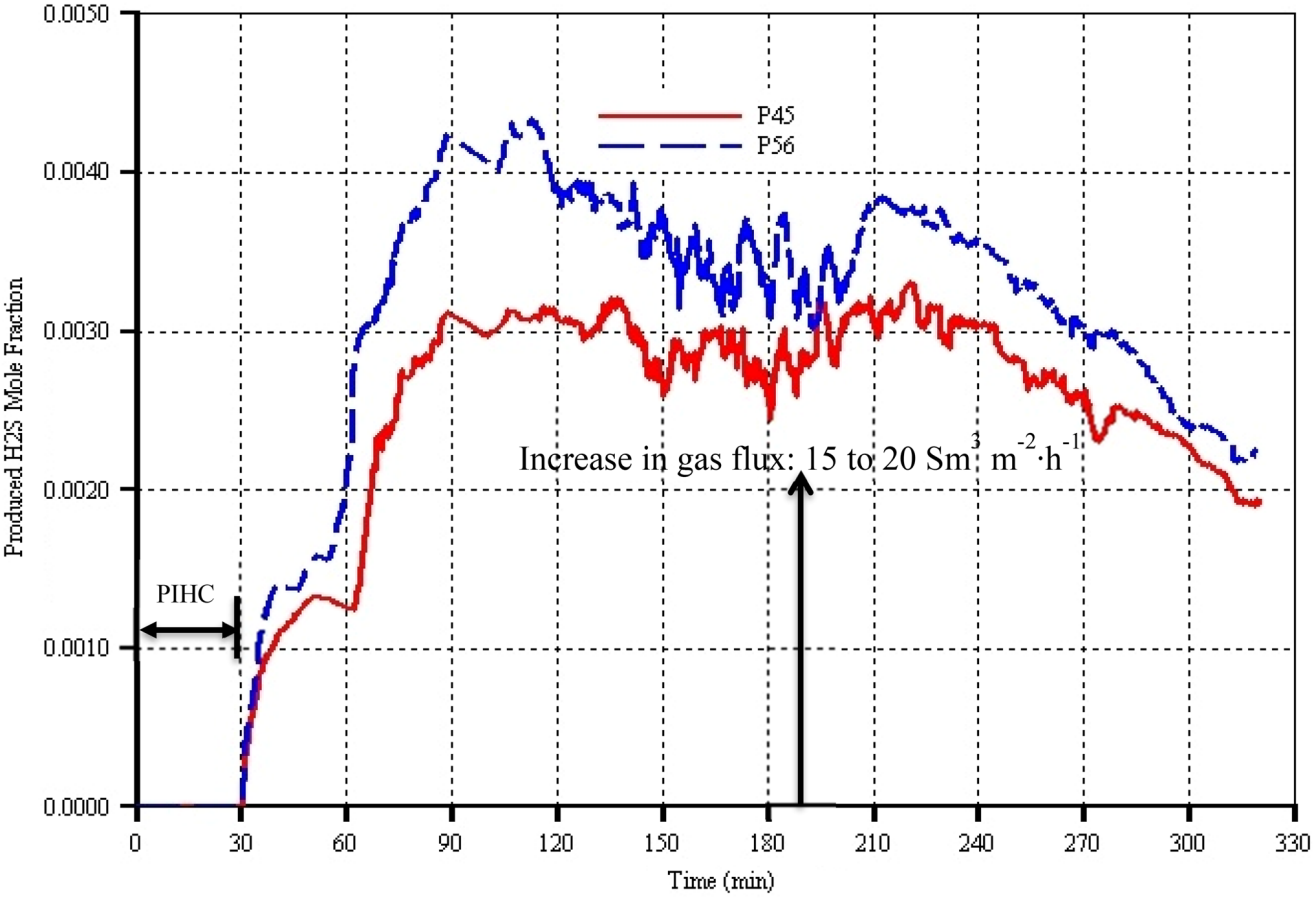
Produced hydrogen sulphide (H_2_S) as function of process time for models P45 and P56 which have catalyst packing porosities of 45% and 56% respectively

The API gravity is used to account for the extent of both thermal cracking and catalytic upgrading of the heavy oils and bitumen. Therefore, larger API gravity relative to that of the native reservoir oil indicates upgrading which will be due to the presence of larger amounts of light components in the produced oil. Since there is no oil production during the 30 min of pre-ignition heating cycle (PIHC), the API gravity in either model is just that of the native reservoir oil, namely 10° API. Immediately when the gas injection began and ignition achieved, model P56 predicted larger API gravity by up to 4° API points in the instantaneously produced oil compared to in model P45 (Fig. 8). Similar to the trends at the early stage of oil production (Fig. 6), the API gravities of both models dropped down rapidly. However, unlike the oil production rates, the sharp rising and sudden dipping of the API gravities in both models took place within 2 to 3 min. After 33 min, the API gravities of the produced oil in the two models rise steadily until they reach, at 90 min, maximums of 25.5° API and 23° API in models P56 and P45 respectively. From 90 min to just before the increase in gas flux (i.e. at 190 min), the API gravities of the produced oil in both models remained constant, but with that of model P56 lying above that of model P45. Thereafter, the API gravities increased momentarily in both models, indicating that lighter components are produced due to both increase in catalytic upgrading (which is reflected by the H_2_S production (see Fig. 9)) and gas-sweeping of already mobilized oil (which is reflected in the oil production rates (see Fig. 6)). From 200 min onwards, the API gravities predicted by both models decreased steadily in similar trends to the oil production rates (see Fig. 6) which are due to the fact that the period of the ‘declining phase’ of oil production rates is entered. This declining in API gravities is also due to the fact that the length of the HP well where the mobilized oil contacts the catalyst for catalytic reactions has decreased dramatically, resulting in a corresponding decrease in the produced H_2_S (Fig. 9). Generally, during the whole THAI-CAPRI process time, the API gravity curve of model P56 lies above that of model P45 by around 0.5 to 2.5 API points. This shows that the larger the catalyst packing porosity, the larger the surface area accessible by the mobilized partially upgraded THAI oil (which is produced due to heat and thermal cracking) which in turn resulted in higher upgrading. To further support the preceding explanation, it is reasoned that the smaller catalyst packing voidage of model P45 must have been filled and blocked by the deposited coke and therefore depriving the mobilized partially upgraded THAI oil the pathways to reach the inner catalyst active sites for the catalytic reactions to take place. This can be seen in Fig. 8 where the hydrogen sulphide formed due to carbon–sulphur bond breakage and carbon–hydrogen and sulphur–hydrogen bonds formations is generally larger in model P56 than in model P45. This new key finding was not shown by the previous study by Hasan and Rigby (2019).

### Produced hydrogen sulphide in the gas stream

**Fig. 10:**
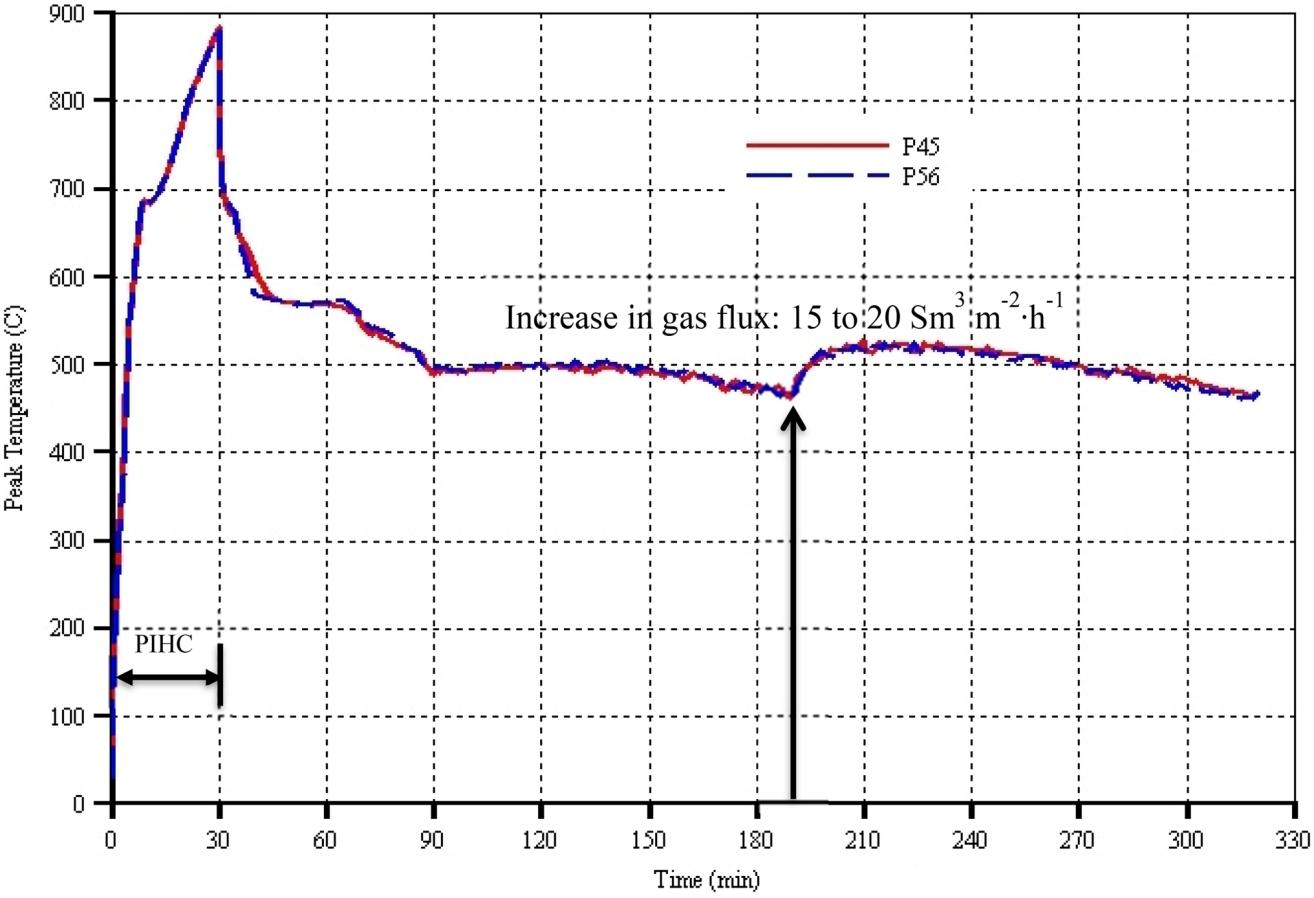
Peak temperature as function of process time for models P45 and P56 which have catalyst packing porosities of 45% and 56% respectively

On gas injection, initiation of the combustion, and the mobilized oil’s and hydrogen’s contact with the catalyst surface, hydrogen sulphide (H_2_S) is shown to be produced (Fig. 9), although at just the beginning of it is formation, it did not follow the same trend as API gravity. This is a key indicator that sulphur–carbon bonds were cleaved and the sulphur was replaced with hydrogen, which resulted in further catalytic upgrading of the mobilized partially upgraded THAI oil. Throughout the process time, more H_2_S is produced in model P56 compared to in model P45. This is caused by the availability of the larger catalyst surface area contacted by the partially upgraded THAI oil, resulting in more upgrading due to the greater extent of the catalytic reactions in model P56. It additionally shows that in model P45 where the catalyst packing porosity is 45%, the deposited coke on the catalyst surface has covered the catalyst to the extent that it has restricted the flow of oil into the inner active sites of the catalyst. Curiously, especially after the first 2 to 3 min of gas injection, there is a similarity in the trends of the API gravity and the concentration of the produced H_2_S (i.e. comparing Figs. 8 and 9), which reinforces the fact that the more sulphur is removed, the higher the quality of the produced oil. The same conclusion is drawn from the hydrodenitrogenation reactions. Prior to the increase in the gas injection flux at 190 min, both models show a steady decrease in the mole fraction of the produced H_2_S which can be attributed to the decrease in the length of the HP well or annular catalyst layer being used for the catalytic reactions as the combustion progresses in a toe-to-heel manner. Increasing the gas flux resulted in a momentary increase in H_2_S mole fraction before the set in of steady decline which is due to the decrease in oil production rate as the control volume is being depleted and is in the oil-rate-declining phase. Interestingly, this also corresponds to the temperature-decreasing phase (Fig. 10).

### Predicted peak temperature

Peak temperature gives the measure of the vigorousness of the combustion front. It indicates the type of oxidation reaction that is dominant, namely low-temperature oxidation (LTO) or high-temperature oxidation (HTO) reaction (Ado, 2020d). Generally, it can be seen that the peak temperatures of both models remain above 470 °C throughout the combustion period, implying that HTO is the dominant oxidation reaction that is taking place (Fig. 10). During the 30 min of PIHC and around 2 min after the start of gas injection and initiation of combustion, the peak temperatures overlapped each other, indicating that they are not affected by the catalyst packing porosity (Fig. 10). This is because the same amount of coke is effectively deposited in either model and that significant quantity of heat did not reach the catalyst bed. Thereafter, there are minor differences between the peak temperature of the two models whereby they differ within about ± 5 °C. At some times, e.g. between 40 and 50 min, and between 230 and 320 min, model P45 predicted slightly high peak temperatures by approximately 5 °C. At all the other times, model P56 predicted slightly high peak temperatures by around 5 °C. On the overall however, although the differences in the peak temperatures have effect on the degree of thermal cracking upgrading, it can be concluded that the catalyst packing porosity has only marginal effect on the predictions of the peak temperatures. This therefore implies that both the combustion reactions, especially HTO, and the thermal cracking reactions in either models are respectively the same and proceeded at roughly the same rates. However, the extent of the catalytic reactions in model P56 is larger than in model P45 which is purely due to the difference in catalyst packing porosities of the two models. This is so since the peak temperatures are only slightly affected by the catalyst packing porosity. Therefore, the only reason for the found differences in the degree of upgrading and the H_2_S production in both models is the difference in the catalyst packing porosities of the two models.

### Produced oxygen mole fraction in the gas stream

**Fig. 11:**
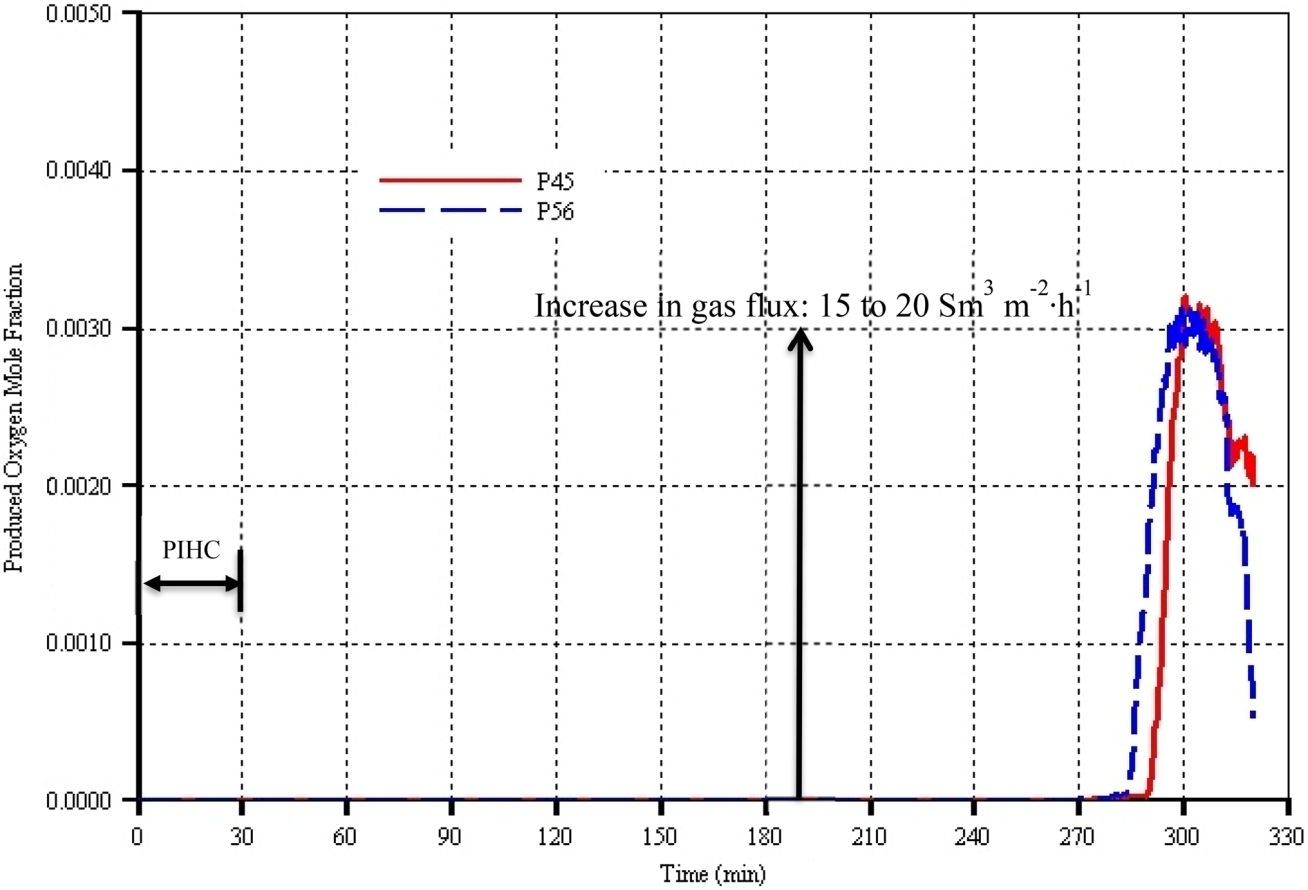
Produced oxygen mole fraction in the gas stream as function of process time for models P45 and P56 which have catalyst packing porosities of 45% and 56% respectively

The concentration of oxygen in the produced gases gives an indication of the stability and efficiency of the combustion. It also allows operators to decide the time at which a producer well should be shut to ensure safe operation. The THAI process is known to be a very stable process in terms of both propagation of combustion front and efficiency of burning. From the start of gas injection and ignition up to 285 min, the produced flue gases in both models do not contain oxygen at all. This is because no oxygen bypasses the high-temperature combustion front. The mole fractions of oxygen in all the two models were zero (Fig. 11) over that period. Oxygen production began at 285 min in model P56, and the maximum it reached was around 0.32 mol% at approximately 300 min before dipping sharply to around 0.05 mol% at the end of the process (i.e. 320 min). Similarly, with the model P45, the maximum concentration of oxygen in the produced gas occurs at around 300 min before it dropped sharply to 0.2 mol% by the end of the process time (Fig. 11). Model P45 lags model P56 by around 5 min. However, the two models predicted identical trends. Conclusively however, the produced oxygen concentration is not particularly affected by the catalyst packing porosity, which is a similar reflection to the peak temperature curves shown in Fig. 10. This also indirectly indicated that in both models, the extent of combustion reactions is the same since they have similar oxygen utilization. In addition, since the two models have similar concentration of produced oxygen, it implies that the extent of thermal cracking is also the same. Therefore, overall, the difference in the degree of upgrading (i.e. API gravity) is totally due to the difference in the catalyst packing porosities. This study has shown that the THAI-CAPRI as a coupled process is very stable and these are regardless of the catalyst packing porosity. Therefore, the key conclusion is that as optimally high as possible catalyst packing porosity should be used since oxygen does not bypass the combustion front. However, future investigations must look into the application of this conclusion at field scale prior to any field deployment.

## Conclusions

In this work, the first ever-detailed investigations of the effect of catalyst packing porosity on the performance of the THAI-CAPRI process were performed through reservoir numerical simulations using computer modelling group (CMG) commercial thermal reservoir simulator, STARS. It is found in this work that over the 290 min of combustion period, slightly more oil (i.e. an additional 0.43% oil originally in place (OOIP)) is recovered in model P56 whose catalyst packing porosity is 56% compared to that recovered in model P45 whose catalyst packing porosity is 45%. In other words, there is a cumulative oil production of 2330 cm^3^ in model P56 compared to the cumulative oil production of 2300 cm^3^ in model P45. Therefore, the catalyst packing porosity has negligible influence on the cumulative volume of oil recovered. It is also found that the peak temperature is only marginally affected by the catalyst packing porosity, thereby implying that regardless of the packing porosity of the catalyst annular layer, the extents of the combustion and thermal cracking reactions are respectively the same. And that means only the extents of the catalytic reactions depend on the catalyst packing porosity. It is also shown that as a result of the dependence of the extents of catalytic reactions on the accessible surface area to reach the inner coke-uncoated catalysts, the larger the catalyst packing porosity, the higher the API gravity and thus quality of the produced oil.

Other findings are that the THAI-CAPRI as a coupled single process is very stable in terms of both combustion front propagation and burning efficiency. This is indicated by the fact that the produced oxygen concentration is independent on the catalyst packing porosity. Another positive advantage shown by this study is that, for every cubic metre (m^3^) of annular space around the HP well, a saving of at least 117 kg of catalyst will be made in terms of catalyst cost if packing porosity of 56% instead of 45% is used. However, for this part, optimal packing porosity should be determined in future studies. As part of suggestions for future studies, full field-scale studies of the THAI-CAPRI process are needed prior to field deployment. This will allow the mechanism via which the mobilized partially upgraded THAI oil will be reaching the catalyst and reacting, since the flow pattern in the field is different from that at experimental scale, to be fully understood and thus to properly allow designing of optimal process operating procedure.
